# Isolation and characterization of plant and human pathogenic bacteria from green pepper (*Capsicum annum* L.) in Riyadh, Saudi Arabia

**DOI:** 10.1007/s13205-013-0136-2

**Published:** 2013-08-14

**Authors:** Samiah H. S. Al-Mijalli

**Affiliations:** Biology Department, College of Sciences, Nora Bent AbdulRahman University, Riyadh, Saudi Arabia

**Keywords:** Green pepper, Pathogenic bacteria, *Klebsiella oxytoca*, *Proteus mirabilis*

## Abstract

Forty-three bacterial isolates in five genera were recovered from naturally infected green pepper fruits (38 samples) showing dark brown, irregular-shaped splotches. The pathogenicity test was performed on healthy green pepper fruits and red colonies were from inoculated fruits showing the same symptoms and the infected area developed into soft rot. Their identification was based on phenotypic characters and sequence of the gene fragment coding 16S rRNA. Of 43 isolates, 10 showing splotches on green pepper fruits belonged to genus *Serratia* on the basis of phenotypic characters. One representative isolate of the genus *Serratia* has been identified by partial 16S rRNA gene sequencing and phylogenetic analysis as belonging to the *Serratia rubidaea* and has the potential to cause spot on green pepper. Eleven phytopathogenic bacterial isolates were also obtained at the same time but did not induce any splotch symptoms on artificially infected green pepper. Five out of 11 bacterial isolates were identified as *Ralstonia* on the basis of biochemical tests. Partial sequencing of 16S ribosomal gene of representative isolate revealed that the isolate is *Ralstonia solanacearum*. The six remaining isolates were related to *Xanthomonas vesicatoria* on the basis of biochemical tests. Twenty-two of opportunistic human pathogens were isolated at the same time and related to *Proteus* and *Klebsiella*. Opportunistic human pathogens did not produce any symptoms on artificially infected green pepper. One representative isolate for each genus was identified as *Klebsiella oxytoca* and *Proteus mirabilis* based on their partial 16S rRNA gene sequences. The virulence of the *S. rubidaea,* the causal agent of green pepper fruits splotches was attributed to the production and secretion of a large variety of enzymes capable of degrading the complex polysaccharides of the plant cell wall and membrane constituents.

## Introduction

The role of fresh fruits and vegetables in nutrition and healthy diet is well recognized and in recent years, many countries have undertaken various initiatives to encourage consumers to eat more of these products. Fruits and vegetables supply much needed vitamins, minerals, and fibers. These play an important role in health through the prevention of heart disease, cancer, and diabetes. The health aspect together with increasing consumer demands for variety and availability, and the changing structure of global trade has led to an increase in trade of fruits and vegetables (Abd-Alla et al. 2011).

Agricultural products can be exposed to microbial contamination through a variety of sources. Although vegetables are good examples of minimally processed foods, there is a high risk of contamination. Since fruits and vegetables are produced in a natural environment, they are vulnerable to contamination by human pathogens. The increased consumption of fruits and vegetables may have unintended consequences with an increase in number of outbreaks. The majority of diseases associated with fresh fruits and vegetables are primarily those transmitted by the fecal–oral route, and therefore, are a result of contamination at some point in the process (De Roever 1998). Therefore good hygienic measures have to be taken during the production from farm to table. The world has seen significant changes in eating habits and consumption of fresh products is increasingly becoming important in the diet of many people, especially reflected in the increased demand for organically produced food. In the production and processing of fresh produce quality and hygiene are the most important criteria for the consumers. Such food products are often eaten raw and, if contaminated with pathogenic bacteria may represent a health hazard to consumers (Bruhn 1995).

Bacterial soft rot is a leading cause of postharvest losses of potatoes (Cappellini et al. 1984), tomatoes (Ceponis et al. 1986), peppers (Ceponis et al. 1987), lettuce (Ceponis et al. 1985), and other fresh fruits and vegetables in the marketplace. It is caused by a group of plant pathogens, harmless to humans, that includes *Erwinia carotovora,* pectolytic *Pseudomonas fluorescens* and *P. viridiflava* (Lund 1983). Pectolytic breakdown of affected tissues results in softening, liquefaction, and exudates that can spread bacteria over commodities in bulk storage or display, contaminate food-handling equipment, and protect bacteria from the environment (Snowdon 1990; Wei et al. 1995).

*Serratia* species were frequently found associated with plants (Grimont et al. 1981). *Serratia marcescens, S. liquefaciens*, and *S. rubidaea* were found in 29, 28, and 11 %, respectively, of vegetable salads served in a hospital in Pittsburgh (Wright et al. 1976). *S. rubidaea,* first described by (Stapp 1940), is an epiphyte on plants. *S. rubidaea* has been isolated from coconuts of Ivory Coast bought in both France and California (Grimont et al. 1981). The relative frequency of *S. rubidaea* in clinical specimens is rare, and there are no data to suggest that the organism is of clinical significance, but clinical significance cannot be totally excluded because of its occurrence in clinical specimens (Farmer et al. 1985).

Sequences of the 16S rRNA gene are generally used as a framework for bacterial classification. Therefore, sequencing of this gene was used as a first identification tool (Garcıa-Martınez et al. 2001). Extracellular enzymes have a number of potential roles in plant disease, including overcoming host defense responses, mobilization of plant cell walls for nutritional purposes, facilitation of movement of bacteria into and between vascular elements, and promotion of bacterial survival on plant material in the soil. Successful management of plant diseases relies on correct diagnosis. Therefore, investigations on pathogenic bacteria and bacterial diseases on the fresh vegetable and fruit plants are economically important. The overall objective of this study was to determine and characterize the bacterial isolates recovered from green pepper fruits spot collected from different vegetable markets and their ability to produce an array of extracellular enzymes capable of degrading the complex polysaccharides of the plant cell wall and membrane constituents.

## Materials and methods

### Sample collection

Naturally infected green pepper *Capsicum annum* L. spots were collected from different vegetable markets in Riyadh, Saudi Arabia. The collected samples (38) were kept in refrigerator until the time of isolation. They were analyzed within 20 h of sampling.

### Bacteriological analysis

Green pepper fruits with spot symptoms were washed with sterilized distilled water, and then treated with 0.5 % solution of hypochlorite (bleach) (Cotter et al. 1985) for 1–2 min to remove the contaminants, rinsed with sterile distilled water and cut into small bits with sterile scalpel. These pieces were immersed in sterilized saline buffer and vortexed strongly. A tenfold dilution series was prepared and 100 μl each of diluted and the undiluted extract was spread (with three replications of each dilution) on yeast dextrose chalk (YDC) medium (Schaad 1988) consisting of 20.0 g/l dextrose, 10.0 g/l yeast extract, and 20.0 g/l CaCO_3_ with 15.0 g/l of agar in 1 l of distilled water. YDC medium was autoclaved for 15 min at 121 °C.

Cultures were incubated for 3–5 days at 30 °C. Discrete colonies were re-streaked onto YDC plates for pure culture isolation. One colony of the purified presumptive pathogen from each sample was selected and maintained on YDC slants at 4 °C for further tests.

### Pathogenicity test

Green pepper fruits were swabbed with 70 % ethanol and washed in sterile water and stabbed with sterile syringe needles at three sites. Inoculations were made by deposition of 5 μl of a bacterial suspension on the upper surface of green pepper fruits. Three fruits were used for each isolate. Inocula were prepared from 48-h-old cultures on nutrient broth medium incubated at 30 °C. Bacterial cells were collected in saline phosphate buffer (pH 7) and adjusted to 10^7^ cfu/ml by turbidity measurement (A600). Inoculated green pepper fruits were kept in closed transparent boxes lined with moist blotting and incubated at 30 °C. All fruits were assessed daily for 7 days to record disease symptoms. The causal agent was recovered from green pepper showing the same symptoms on YDC. All bacterial isolates were tested for their ability to produce any symptoms.

### Biochemical characterization of bacterial isolates

Bacteriological characteristics of the isolates were examined by using the methods to include: Gram stain, Ryu’s test, colony color, oxidase reaction, arginine dihydrolase, nitrate reduction, utilization of carbohydrates, Levin formation, catalase test, gelatin hydrolysis, starch hydrolysis, esculin and Tween 20, as previously described by Bergey’s Manual (Brenner et al. 2005).

### Sequencing of 16S rRNA gene

DNA was extracted by boiling a small amount of a pure culture plate colony in DNAse- and RNAse-free water (Invitrogen, Carlsbad, CA). For the sequencing of the partial 16S rRNA gene fragments polymerase chain reaction amplifications were performed with universal bacterial primers (Lane 1991) [27f (9–27) GAGTTTGATCMTGGCTCAG and 1492r (1492–1510) ACGGYTACCTTGTTACGACTT] in a total volume of 25 μl. The reaction mixture contained 2.5 μl PCR-buffer, 2.0 μl MgCl_2_ (25 mM), 2.5 μl dNTPs (2 mM), 0.5 μl of each primer, 15.7 ml RNAse- and DNAse-free water, 0.2 μl BSA (20 mg/ml), 0.1 μl Taq polymerase (5 U μl^−1^) (all MBI Fermentas, St. Leon Rot), and 1 μl of the DNA extract. The reaction was performed using an Eppendorf Ag 22331 Authorized Thermal Cycler (Hamburg, Germany) with an initial denaturation step at 95 °C for 3 min, followed by 29 cycles of denaturation (95 °C for 45 s), annealing (57.3 °C for 20 s) and extension (72 °C for 2 min). The PCR was completed with a terminal extension step for 4 min at 72 °C. PCR products were purified (PCR Purification Kit250, QiaQuicks, Qiagen, Hilden) and quantified photometrically (Ultrospec 4000, Amersham Biosciences, Freiburg). Purified PCR products were cycle sequenced in both directions with the same forward and reverser primers using an Applied Biosystems 3730X-1 DNA Analyzer (Fast Smack Inc. Division DNA synthesis, Kanagawa, Japan). The sequence reads were edited and assembled using BioEdit version 7.0.4 (http://www.mbio.ncsu.edu/BioEdit/bioedit.html) and clustalW version 1.83 (http://clustalw.ddbj.nig.ac.jp/top-e.html). BLAST searches were done using the NCBI server at http://www.ncbi.nlm.nih.gov/blast/Blast.cgi. Phylogenetic tree derived from 16S rRNA gene sequence was generated in comparison to 16S rRNA gene sequences from seven different standard bacterial strains obtained from Genbank (Lane 1991). Gene sequences that had been determined were phylogenetically analyzed using the ARB software package (Sanger et al. 1977). New sequences not included in the used ARB database were added from public databases (http://www.ncbi.nlm.nih.gov/BLAST) using BLASTN search to assign the closed relatives. The ARB_Edit tool was used for automatic sequence alignment and checking and correcting the alignment afterwards. Neighbor-joining algorithms (Drancourt et al. 2000) were used for calculating the trees.

### Enzymes’ production by *Serratia rubidaea*

*Serratia rubidaea* isolate was screened for its ability to elaborate hydrolytic enzymes such as lipase enzyme, protease enzyme, polygalacturonase enzyme and alkaline phosphatase enzyme. Bacteria were grown in 50 ml of liquid medium in an Erlenmeyer flask (250 ml) containing (g/l): MgSO_4_·7H_2_O 0.2, K_2_HPO_4_ 2.0, KH_2_PO_4_ 2 and casein 10 (pH 8) (Chakraborty and Srinivasan 1993). The basal medium for lipase production consisted of (g/l): bacteriological peptone 15.0, yeast extract 5.0, NaCl 2.0, MgSO_4_ 0.4, K_2_HPO_4_ 0.3, KH_2_PO_4_ 0.3, and olive oil 10.0 ml for lipase induction (Baharum et al. 2003). The basal medium for pectinase production consisted of (g/l): pectin 4, yeast extract 2, NH_4_Cl 1, MgSO_4_ 0.5 (Gomes et al. 1992). Cultures were incubated in an orbital shaking incubator for 36 h at 150 rpm and 37 °C. The culture broth was then centrifuged at 8,000 rpm [may be better in *g* (relative centrifuge force)] to remove cells. The clear supernatant was collected for enzymes assay.

### Enzymes assay

Protease activity was assayed by a modified method of Ohara-Nemoto et al. (1994). The reaction was initiated by addition of 1 ml supernatant to 2 ml of reaction mixture containing 2.7 mg of casein per ml in 50 mmol Tris–HCl (pH 8.0) which had been prewarmed at 37 °C. After incubation at 37 °C for 1 hour, the reaction was stopped by addition of 0.5 ml of 15 % ice-cold trichloroacetic acid. The reaction mixture was held on ice for 15 min and then centrifuged; 5 ml of sodium carbonate solution (500 mmol) was added to the reaction mixture followed by 1 ml of Folin and Ciocalteu’s phenol reagent (dilute 10 ml of Folin and Ciocalteu’s phenol reagent, to 40 ml with distilled water). The soluble peptide in the supernatant fraction was measured with tyrosine as the reference compound. The absorbance at 660 nm of the sample was measured using a spectrophotometer (UNICO UV-2100, USA). One unit of enzyme activity was defined as the amount of enzyme that releases 1 μg of tyrosine per min under the assay conditions. Controls containing autoclaved enzymes instead of active enzymes were used. Lipase activity was measured by universal titrimetric method (Fadıloğlu and SÖylemez 1997).

Polygalacturonase was determined according to the method of Gomes et al. (1992). One unit of activity is defined as that amount of enzyme which catalyzes the release of 1 μmol of reducing groups per min and expressed as U/ml.

Alkaline phosphatase of bacterial culture supernatant was determined by standard assay procedure using alkaline phosphatase kit (ELI Tech, SEPPIM S.A.S.-Zone industrielle-61500 SEES France). One alkaline phosphatase unit was defined as the amount of enzyme which liberates 1 μmol of *p*-nitrophenol as a result of hydrolysis of *p*-nitrophenylphosphate (pNPP) in 1 min (Abd-Alla 1994).

### Effects of temperature on enzyme production and stability

The effect of temperature on lipase, protease, polygalacturonase, and alkaline phosphatase enzymes production was determined by incubating the culture flasks with different temperature regimes (10, 20, 30, and 40 °C). For determining thermal stability, the enzyme was pre-incubated at different times ranging from 10 to 70 min at maximum temperature (45 °C) and residual activity was measured under standard assay conditions.

## Results

### Bacterial isolates

A total of 38 samples were randomly taken from different vegetable markets of Riyadh. Forty-three bacterial isolates were obtained from naturally green pepper spot fruits. Four different bacterial colonies were observed on YDC medium. These colonies were distinguished into one type of yellow, two types of white mucoid and one type red.

### Pathogenicity test

The pathogenicity test was performed on healthy green pepper fruits and red colonies were re-isolated from inoculated fruits showing splotches. Symptoms appeared as dark brown, irregular-shaped splotches and the infected area developed into soft rot. The other bacterial isolates of yellow and white colonies did not show any spot symptoms on green pepper fruits.

### Phenotypic and genotypic characterization of the bacterial isolates

**Fig. 1:**
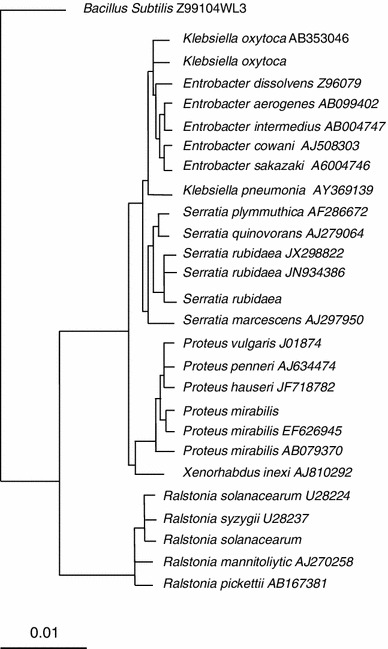
*Phylogenetic tree* indicates the phylogenetic relationship of the isolated strains. Isolates are indicated in *bold*. A neighbor-joining tree was calculated using partial 16S rRNA gene sequences (1,362 bp) and a frequency filter included in the ARB software package. *Bacillus subtilis* was used as out-group. The *scale bar* indicates 10 % estimated sequence difference. Accession numbers of the National Centre for Biotechnology Information (NCBI) database of each strain are given in *brackets*

Ten out of 43 bacterial isolates showing spot on green pepper fruits were further characterized using comprehensive range of phenotypic test (Table 1). The isolates might be belonging to the genus of *Serratia* on the basis of their phenotypic characterization. One representative isolate of the genus *Serratia* was subjected to the partial 16S rRNA gene sequences of 612 base pairs. The selected isolates had 16S rRNA gene sequence with 99 % similarity to the closet sequence of *S. rubidaea* AB004751 in GenBank. Eleven phytopathogenic bacterial isolates were also recovered at the same time. Five out of eleven bacterial isolates were identified as *Ralstonia* on the basis of biochemical tests. One representative isolate was chosen for further identification using phylogenetic analysis of 16S rRNA gene sequences as the gold standard. The partial 16S rRNA gene sequence of 630 base pairs of the representative isolate had a sequence with 99 % similarity to *Ralstonia solanacearum* U28224. A phylogenetic tree was constructed from a multiple sequences alignment of 16S rRNA gene sequences (Fig. 1). The other six isolates were related to *Xanthomonas vesicatoria* on the basis of phenotypic characters.

**Table 1:** Biochemical characterization test of bacterial genera isolated from green pepper fruits

Characteristics	Group 1	Group 2	Group 3	Group 4	Group 5
Phenotypic classification	*Serratia*	*Ralstonia*	*Xanthomonas*	*Klebsiella*	*Proteus*
Fluorescent on king’s B	**–**	**–**	**–**	**–**	**–**
Growth on cetrimide	**–**	**–**	**–**	**–**	**–**
Gram’s staining	**–**	**–**	**–**	**–**	**–**
KOH solubility	**+**	**+**	**+**	**+**	**+**
Cytochrome C oxidase	**–**	**+**	**–**	**–**	**–**
Nitrate reductase	**+**	**+**	**–**	**+**	**+**
Catalase test	**+**	**+**	**+**	**+**	**+**
Gelatin hydrolysis	**+**	**+**	**+**	**–**	**–**
Casein hydrolysis	**+**	**+**	**+**	**–**	**–**
Starch hydrolysis	**–**	**–**	**–**	**+**	**+**
Arginine dehydrogenase	**–**	**–**	**–**	**+**	**–**
Urease test	**–**	**+**	**–**	**–**	**+**
H_2_S production	**–**	**–**	**–**	**+**	**+**
Esculin test	**+**	**+**	**+**	**+**	**–**
Voges–Proskauer	**–**	**–**	**–**	**–**	**–**
Carbon source utilization					
l-Arabinose	**–**	**–**	**+**	**+**	**–**
d-Cellobiose	**+**	**–**	**+**	**+**	**–**
d-Fructose	**–**	**+**	**+**	**+**	**–**
Citrate	**+**	**–**	**–**	**+**	**–**
d-Alanine	**+**	**–**	**–**	**+**	**+**
d-Sorbitol	**–**	**+**	**–**	**+**	**+**
d-Galactose	**+**	**–**	**–**	**+**	**–**
Glycerol	**+**	**+**	**+**	**–**	**+**
Glucose	**+**	**–**	**+**	**+**	**+**
Lactose	**+**	**–**	**–**	**+**	**+**
Maltose	**+**	**+**	**+**	**+**	**–**
Mannitol	**+**	**+**	**+**	**+**	**+**
Growth at 4 °C	**–**	**–**	**–**	**–**	**–**
Growth at 37 °C	**+**	**+**	**+**	**+**	**+**
Growth at 41 °C	**+**	**–**	**+**	**+**	**+**

Symbols  – and  + meaning negative and positive

Other 22 non-phytopathogenic bacterial isolates were recovered at the same time from green pepper fruits. Ten out of 22 belonged to the genus *Klebsiella* on the basis of biochemical activities. The 16S rRNA gene sequence of 571 bp of the representative isolate was aligned with other 16S rRNA gene sequence using ARB software package to demonstrate the relatedness of the isolate to other major groups. Sequence from the analyzed isolate shared 98 % similarity to known strain of *Klebsiella oxytoca* AB353048 in GenBank database as supported by phylogenetic tree. The twelve remaining isolates were subjected to varieties of biochemical tests to determine the phenotypic traits and enable their identification. Data presented in Table 1 demonstrated that the isolates clearly belong to the genus of *Proteus.* The phenotype-based identification was confirmed by phylogenetic analysis. Comparison between 16S rRNA gene sequence of the chosen isolates of genus *Proteus* and 16S rRNA gene sequences of GenBank database was made by using BLASTN search analysis. Sequencing of 16S rRNA genes of the chosen isolate had 16S rRNA gene with 99 % nucleotides identity to that of *Proteus mirabilis* EF626945 available in GenBank database. The phylogenetic tree was inferred from 16S rRNA sequence data by the neighbor-joining method (Fig. 1). The tested analyzed isolate was identified as *P. mirabilis* belonging to the family Enterobacteriaceae.

### Effect of temperature on enzymes production

**Fig. 2:**
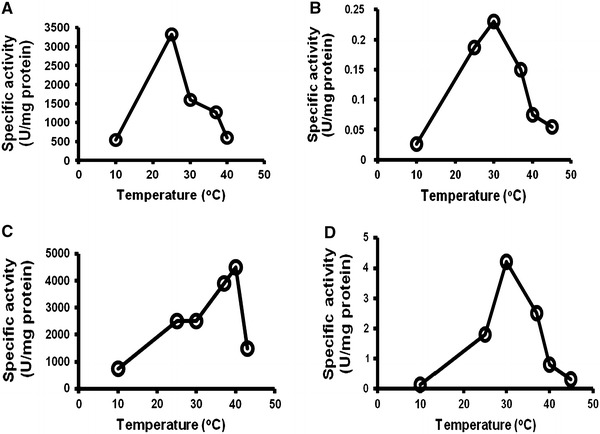
Effect of temperature on enzymes production of *Serratia rubidaea.***a** Lipase enzyme, **b** protease enzyme, **c** polygalacturonase enzyme, **d** alkaline phosphatase enzyme

*Serratia rubidaea* has the ability to produce the extracellular enzyme which was thought to play an important role in host infection. Lipase, protease, polygalacturonase and alkaline phosphatase enzymes were detected in *S. rubidaea* isolates. The optimum temperature for lipase production by *S. rubidaea* was 25 °C (Table 2; Fig. 2). It is obvious from the results in Fig. 2 that 30 °C temperature was generally more favorable for protease production by *S. rubidaea*. The optimum temperature for polygalacturonase production by *S. rubidaea* was 40 °C. The results presented in Fig. 2 revealed that the highest production of alkaline phosphatase by *S. rubidaea* was achieved at 37 °C (Fig. 2; Table 2).

**Table 2:** Effect of temperature on enzymes production of *Serratia rubidaea* lipase enzyme, protease enzyme, polygalacturonase enzyme, and alkaline phosphatase enzyme

Temperature	Lipase	Protease	Polygalacturonase	Phosphatase
10	550	0.026	750	0.15
25	3,320	0.187	2,500	1.8
30	1,600	0.23	2,500	4.2
37	1,265	0.15	3,900	2.5
40	600	0.075	4,500	0.8
45	550	0.055	1,500	0.32

Data expressed as U/mg protein

### Thermal stability of produced enzymes

**Fig. 3:**
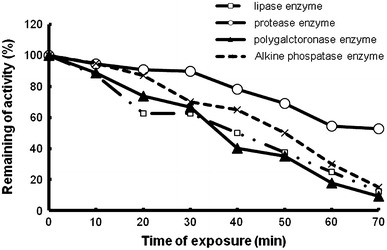
Thermal stability of enzymes produced by *Serratia rubidaea* at 45 °C

Thermal stability was investigated by incubating the enzymes produced by *S. rubidaea.* Lipase enzyme of *S. rubidaea* exhibits the highest thermal stability with 87 % of maximum activity remaining after 10 min, but 50 % of the initial activity retained at 45 °C for 40 min (Fig. 3). Protease enzyme of *S. rubidaea* exhibits the highest thermal stability with 94 % of maximum activity remaining after 10 min, but 87 % of the initial activity retained at 45 °C for 40 min (Fig. 3). The optimum temperature for polygalacturonase produced by *S. rubidaea* was 40 °C. At 45 °C, polygalacturonase enzyme of *S. rubidaea* exhibits the highest thermal stability with 88 % of maximum activity remaining after 10 min, however, 69 % of the initial activity retained for 40 min. Alkaline phosphatase enzyme of *S. rubidaea* exhibits the highest thermal stability with 93 % of maximum activity remaining after 10 min, but 65 % of the initial activity retained at 45 °C for 40 min.

## Discussion

Phenotypic and genotypic techniques were used for identification and allowed us to infer the phylogeny of 43 bacterial isolates recovered from green pepper fruits, distributed along five genera (*S. rubidaea, X. vesicatoria, R. solanacearum, K. oxytoca* and *P. mirabilis*) and comprising three bacterial families. Most pathogenic microbes must access the plant interior, either by penetrating the leaf or root surface directly or by entering through wounds or natural openings, such as stomata or leaf hydathodes (Kroupitski et al. 2009).

Plant pathogens may grow briefly on or in wounded tissue before advancing into healthy tissue. Injection of *Salmonella* into the tomato stem may introduce the pathogen into xylem, which has the principal role of transporting water and nutrients from the root to the extremities of the plant. Additionally, in the secondary xylem, the axial and ray parenchyma store nutrients and water (Blostein 1991) which sustain viability of plants and, possibly, promote survival of pathogenic bacteria. The presence of epiphyseal flora within tissue of fruits and vegetables through various pathways was reported by (Samish et al. 1962). By examining eight internal locations of green pepper fruits, they observed that bacteria are unevenly distributed in the fruit, and entry may be from the stem scar tissue through the core and into the endocarp. This study suggested that some epiphyseal flora might reach internal tissue of tomatoes through natural apertures because of their small size and motility. It may be that bacteria enter fruit tissue more readily in the early stages of fruit development, at a time when various channels are not yet covered by corky or waxy materials (Samish et al. 1962). Broken trichomes on young fruits represent another site of entry of microorganisms. Guo et al. 2001 reported that green pepper fruits, stem and flowers are possible sites at which *Salmonella* may attach and remain viable during fruit development, thus serving as routes or reservoirs for contaminating ripened fruit.

*Klebsiella oxytoca* is an opportunistic pathogen involved in nosocomial infections and antibiotic-associated diarrhea and hemorrhagic colitis (Högenauer et al. 2006; Gorkiewicz 2009). *K. oxytoca* can cause serious infections, bacteremia, and septic shocks in immunocompromised individuals (Al-Anazi et al. 2008). *P. mirabilis* is a common cause of urinary tract infection (Zunino et al. 2000). Recent studies have shown that enteric bacteria can colonize the interiors of plants (Tyler and Triplett 2008; Berg et al. 2005). Endophytic colonization was shown to result from root infection or contamination of seeds (Tyler and Triplett 2008). The extent of endophytic colonization is determined by the genetic background of both the microbe and the host plant (Tyler and Triplett 2008).

*Serratia rubidaea* was the most prevalent bacteria recovered from naturally infected green pepper fruits in this study, representing 85 % of the total. The pathogenicity test was performed on healthy green pepper fruits and red colonies were reisolated from inoculated fruits showing splotches.

The habitats of *S. rubidaea* are not perfectly known. *S. rubidaea* has been repeatedly isolated from coconuts bought in France (originating mostly from Ivory Coast) and in California (Grimont et al. 1981). It has been isolated from coconuts and from vegetable salads, but it has not been reported from water, insects, small mammals, or animal territories (Grimont and Grimont 1991).

*Serratia* species are important in plant and food microbiology because not only are they involved in food spoilage but also they are opportunistic pathogens that can cause various diseases in humans, animals, and plants. They have been isolated from coconuts and vegetable salads, but not reported from water, insects, small mammals, or animal territories (Grimont and Grimont 1991).

The virulence of the plant pathogen *S. rubidaea* is dependent on the production and secretion of a large variety of plant cell wall-degrading enzymes and membrane constituents, including polygalacturonase, lipase, protease and alkaline phosphatase. The activity of the type of induced enzyme may be influenced by environmental factors. Their activity may be significantly diminished or destroyed by a variety of physical or chemical agents resulting in a loss of the functions performed by the enzymes. A characteristic feature of many phytopathogenic organisms is their ability to produce an array of enzymes capable of degrading the complex polysaccharides of the plant cell wall and membrane constituents. These enzymes are usually produced inductively and are extracellular, highly stable and present in infected host tissue (Bateman and Basham 1976). A major virulence factor in onion pathogenicity is the presence of a polygalacturonase enzyme involved in tissue maceration that is encoded by the plasmid-borne pehA gene (Gonzalez et al. 1997). In conclusion, these bacteria isolated in current study may be pathogenic for humans and could be a threat to human health in food.
